# Ultra-low-dose CT for attenuation correction: dose savings and effect on PET quantification for protocols with and without tin filter

**DOI:** 10.1186/s40658-023-00585-0

**Published:** 2023-10-20

**Authors:** Natalie Anne Bebbington, Kenneth Boye Christensen, Lone Lange Østergård, Paw Christian Holdgaard

**Affiliations:** 1 Siemens Healthcare A/S, Borupvang 9, 2750 Ballerup, Denmark; 2 Department of Nuclear Medicine, Lillebaelt Hospital - University Hospital of Southern Denmark, Beriderbakken 4, 7100 Vejle, Denmark

**Keywords:** PET, CT, Attenuation correction, Ultra-low, Dose, Quantification, Tin filter

## Abstract

**Background:**

Ultra-low-dose (ULD) computed tomography (CT) scans should be used when CT is performed only for attenuation correction (AC) of positron emission tomography (PET) data. A tin filter can be used in addition to the standard aluminium bowtie filter to reduce CT radiation dose to patients. The aim was to determine how low CT doses can be, when utilised for PET AC, with and without the tin filter, whilst providing adequate PET quantification.

**Methods:**

A water-filled NEMA image quality phantom was imaged in three configurations with ^18^F-FDG: (1) water only (0HU); (2) with cylindrical insert containing homogenous mix of sand, flour and water (SFW, approximately 475HU); (3) with cylindrical insert containing sand (approximately 1100HU). Each underwent one-bed-position (26.3 cm) PET-CT comprising 1 PET and 13 CT acquisitions. CT acquisitions with tube current modulation were performed at 120 kV/50 mAs-ref (reference standard), 100 kV/7 mAs-ref (standard ULDCT for PET AC protocol), Sn140kV (mAs range 7–50-ref) and Sn100kV (mAs range 12–400-ref). PET data were reconstructed with μ-maps provided by each CT dataset, and PET activity concentration measured in each reconstruction. Differences in CT dose length product (DLP) and PET quantification were determined relative to the reference standard.

**Results:**

At each tube voltage, changes in PET quantification were greater with increasing density and reducing mAs. Compared with the reference standard, differences in PET quantification for the standard ULDCT protocol for the three phantoms were ≤ 1.7%, with the water phantom providing a DLP of 7mGy.cm. With tin filter at Sn100kV, differences in PET quantification were negligible (≤ 1.2%) for all phantoms down to 50mAs-ref, proving a DLP of 2.8mGy.cm, at 60% dose reduction compared with standard ULDCT protocol. Below 50mAs-ref, differences in PET quantification were > 2% for at least one phantom (2.3% at 25mAs-ref in SFW; 6.4% at 12mAs-ref in sand). At Sn140kV/7mAs-ref, quantification differences were ≤ 0.6% in water, giving 3.8mGy.cm DLP, but increased to > 2% at bone-equivalent densities.

**Conclusions:**

CT protocols for PET AC can provide ultra-low doses with adequate PET quantification. The tin filter can allow 60–87% lower dose than the standard ULDCT protocol for PET AC, depending on tissue density and accepted change in PET quantification.

## Background

Positron emission tomography (PET) forms images of patient disease activity at the molecular level, by imaging radiopharmaceutical distribution in the patient. The radionuclide decays by positron emission, with the positron subsequently annihilating, generating two back-to-back gamma photons at 511 keV, which are detected on opposite sides of the PET gantry. A virtual line of response is drawn between these two detectors, and the event assumed to originate along that line. However, the detection process relies on both photons reaching the detectors without being attenuated. For this reason, a greater proportion of photons originating from the edge of the patient or organ are detected, compared with those from the centre. This results in artefacts in the PET images, whereby the signal is apparently increased at the edges and reduced towards the centre. Compensating for these PET photon losses through the process of attenuation correction (AC) improves PET image quality and quantification [1] and is considered essential for clinical reading.

Computed tomography (CT) images acquired in hybrid PET-CT examinations provide an image volume representing the radiodensity of the patient’s tissue in each voxel, using the Hounsfield unit (HU). This information is transformed into a PET attenuation map (μ-map): a volume dataset in which each voxel represents the linear attenuation coefficient (LAC) for 511 keV photons, according to the tissue density and distance of travel [2]. A bilinear calibration curve is used to transform CT HU to LAC, in the process described by Carney et al. [2]. The μ-map is used to compensate each voxel in the PET image for its photon losses through attenuation, during attenuation-corrected PET image reconstruction. In addition to AC, the CT scan may also be performed for additional lesion localisation, characterisation or fully diagnostic purposes, with required CT image quality and therefore radiation dose increasing through these clinical purposes [3].

PET-CT is one of the main contributors to the total radiation burden of medical imaging [4]; thus, effort should be made to optimise dose from both PET and CT aspects. CT scans performed for the sole purpose of PET AC have provided the greatest variation in doses between facilities, suggesting great scope for optimisation of such protocols [3]. In AC-only CT scans, good image detail is not required, and high noise can be tolerated; hence, ultra-low-dose (ULD) CT scans can be used to fulfil this purpose [5]. However, such low CT exposure settings for PET AC protocols have seldom been implemented in clinical practice [3], despite Xia et al. and Fahey et al. reporting that adequate PET AC can be achieved with as little as 5 mAs with a tube voltage of 120 kV [5, 6].

CT systems are equipped with an aluminium bowtie filter with two important functions: firstly, to filter out low-energy X-rays which will just be absorbed by the patient and not contribute to the images, to reduce patient dose. Secondly, the bowtie filter shapes the X-ray beam to compensate for different path lengths travelled by the photons through the patient, to provide a uniform signal. Biograph PET-CT systems (Siemens Healthineers, Knoxville, TN, US) provide an ULDCT protocol for PET AC with this standard filtration, using a tube voltage of 100 kV and reference mAs of 7. A tin (Sn) filter has recently become available on Biograph mCT, Biograph Vision, and Biograph Vision Quadra PET-CT systems utilising a Definition Edge CT subsystem (Siemens Healthineers, Forchheim, Germany). Compared with standard filtration, the addition of the tin filter removes a greater proportion of lower energy photons from the beam which would otherwise be absorbed by the patient. A higher mAs is then used to compensate for increased noise, but overall, the required image quality can be achieved with a lower dose.

The tin filter has been shown to reduce radiation doses substantially for standalone CT examinations performed without contrast enhancement, for imaging of bone [7–9], lung nodules [10, 11], abdominal imaging [12] and coronary calcium scoring [13, 14], amongst others. The tin filter could potentially allow large dose reductions for ULDCT scans for PET AC compared with the standard ULDCT protocol, and for localisation/characterisation level scans performed in PET-CT, but the effect of the tin filter on PET quantification should be ascertained, since the tin filter increases average beam energy, thereby impacting CT HUs. The effects of tube voltage changes on PET quantification have been explored previously and found to provide negligible difference when tube voltage was appropriate for patient size at soft tissue equivalent density [15], but this study was made prior to tin filter availability in PET-CT, nor did it consider bone equivalent densities.

The aims of this phantom study were to determine the impact of the tin filter on PET quantification, and to establish how low CT doses can be for CT scans performed in ULDCT for PET AC, including high-density tissue equivalent with and without the tin filter, whilst providing adequate PET quantification.

## Methods

### Phantom configurations

The National Electrical Manufacturers Association (NEMA) International Electrical Commission (IEC) PET Body Phantom, more commonly referred to as the NEMA image quality phantom, which represents a standard sized adult abdomen, was imaged in three configurations. Firstly, with a water and Fluorine-18 labelled fluorodeoxyglucose (^18^F-FDG) solution (62 MBq, 6.35 kBq/ml) without cylindrical insert, providing a density of 0 HU, representing attenuation in soft tissue. In the second configuration, the cylindrical insert (inner diameter 4.4 cm) was included, filled with a homogenous mixture of sand, flour, water and ^18^F-FDG (SFW) (5.3 MBq, 14.48 kBq/ml) at approximately 475 HU representing trabecular bone, with non-active water background. Lastly, the cylindrical insert was filled with a homogenous mixture of sand and ^18^F-FDG (26 MBq, 71.57 kBq/ml), at approximately 1100 HU representing cortical bone, with non-active water background. The inner lining of the cylinder was protected by a high-density polyethylene bag.

### PET-CT acquisition and reconstruction

Each of the three phantom configurations underwent a one-bed-position PET-CT scan on a Biograph Vision 600 with 26.3 cm axial field-of-view (FOV) with Definition Edge 128 slice CT, in which 1 PET and 13 CT acquisitions were made.

CT acquisitions used CARE Dose 4D tube current modulation, in which reference mAs settings were defined for a standard-sized patient, with spine organ characteristic. Scans were performed at: 120 kV with 50 mAs-ref (reference standard); 100 kV with 7 mAs-ref (Siemens Healthineers standard ULDCT protocol for PET AC); Sn140 kV with 7, 12, 25 and 50 mAs-ref; and Sn100 kV with 12, 25, 50, 100, 200, 300 and 400 mAs-ref. On Siemens Healthineers PET-CT systems, mAs values also incorporate the pitch factor, such that:

$${\text{Effective}}\;{\text{mAs}} = [{\text{tube}}\;{\text{current}}\;\left( {{\text{mA}}} \right)*{\text{rotation}}\;{\text{time}}\;\left( {\text{s}} \right)]/{\text{pitch}}\;{\text{factor}}$$

Choice of mAs-ref range at each tube voltage was informed by preliminary phantom work studying the doses delivered and artefacts generated by each kV/mAs-ref combination. Given that the aim of this work was to validate that CT dose reductions still provided adequate PET quantification for AC only CT protocols, kV/mAs-ref combinations which were known to provide higher doses than those already in clinical use without the tin filter were not used.

CT reconstructions were made with Advanced Modeled Iterative Reconstruction (ADMIRE) with strength 3, a Br38 kernel and extended FOV.

**Fig. 1:**
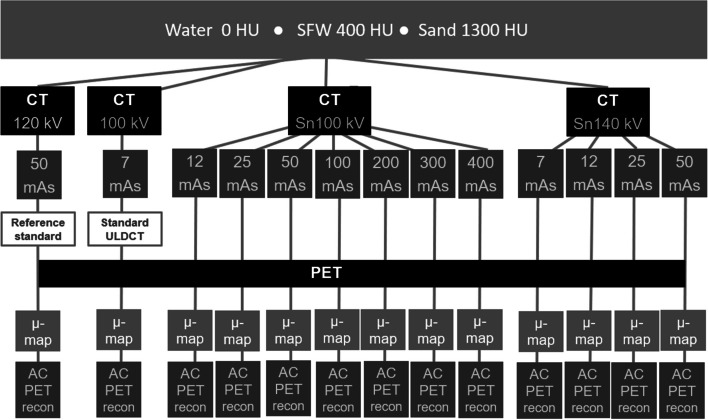
Overview of the CT and PET datasets generated in this study. AC = attenuation correction, HU = Hounsfield unit, mAs = milliampere seconds, SFW = sand + flour + water, ULDCT = ultra-low-dose CT

PET scans used a 10-min acquisition time. For each phantom configuration, 13 AC PET reconstructions were made with the iterative + time-of-flight (TOF) method (4 iterations, 5 subsets, 4 mm Gaussian post filter, matrix 440, zoom 1, relative scatter correction), utilising the μ-maps provided by each of the 13 different CT scans. Figure 1 provides an overview of the data generated by the study.

### Image and results analysis

**Fig. 2:**
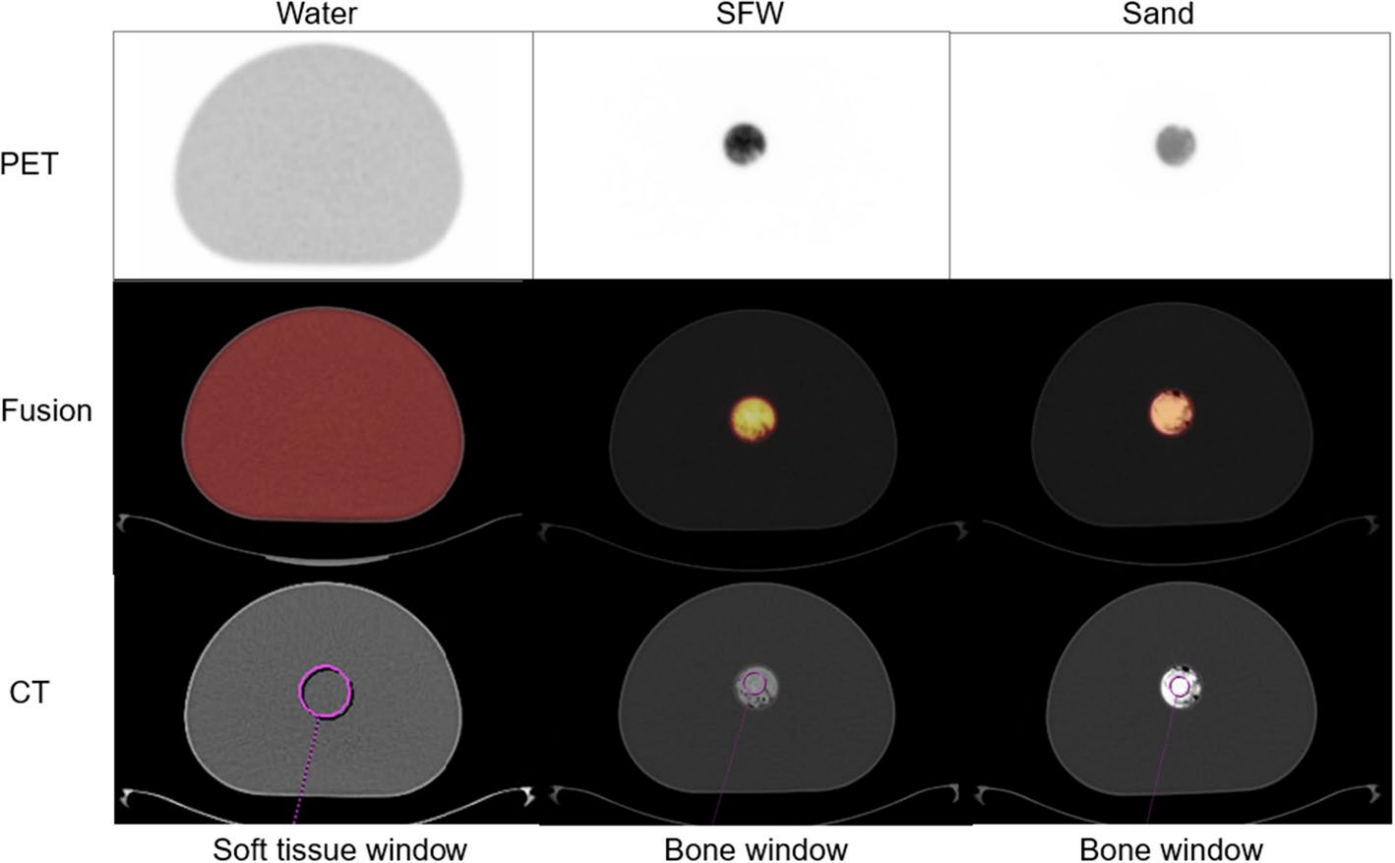
Placement of VOIs for measurement of CT HU and PET activity concentration measurements in each phantom. SFW = sand + flour + water

Image analysis was performed in the MM Oncology workflow in *syngo*.via software version VB50 (Siemens Healthineers, Erlangen, Germany), and results analysis performed in Microsoft® Excel® for Microsoft 365 MSO. For each phantom, a spherical volume of interest (VOI) of 5.0 cm^3^ (sand), 7.2 cm^3^ (SFW) or 82 cm^3^ (water) was assigned to each of the 13 CT datasets and corresponding PET reconstructions using the copy-paste function, and CT HU and PET activity concentration was measured within the VOIs shown in Fig. 2. The chosen VOI size for each configuration was dependent on the evenness of filling of the radioactive mixture in the cylinder, trying to avoid non-active air gaps in the VOI. Differences in PET quantification were calculated relative to the reference standard 120 kV/50 mAs-ref dataset, which had exposure settings typical of localisation/characterisation level CT in FDG PET-CT [3] and was free from significant artefacts caused by low photon fluence. A ± 2% difference in PET quantification compared with the reference standard was considered negligible. Absolute differences in CT HU from the reference standard were calculated, by subtracting the HU of the reference dataset from the HUs of all other exposure settings.

CT dose length product (DLP) was recorded for each CT scan. Doses for the 100 kV/7 mAs-ref standard ULDCT protocol were expressed relative to that of the 120 kV/50 mAs-ref reference standard, and the doses for the tin filter protocols expressed relative to the standard ULDCT protocol.

## Results

### Standard ULDCT protocol for PET AC (100 kV/7 mAs-ref)

**Fig. 3:**
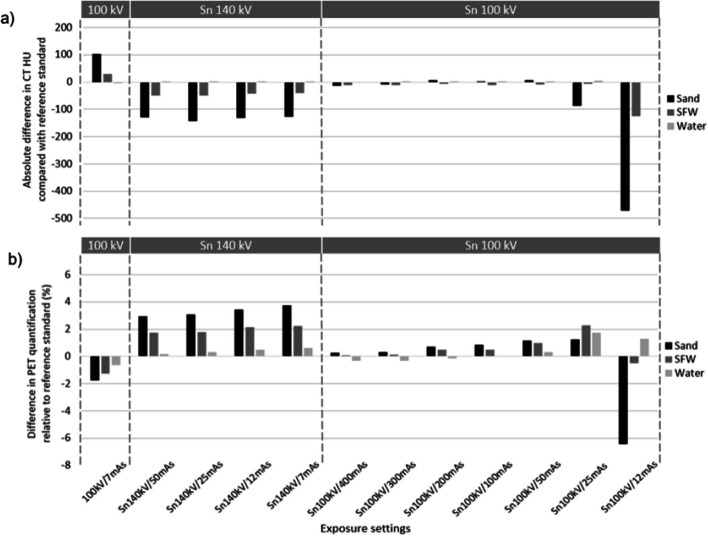
Absolute differences in CT HU (**a**) and relative differences in PET activity concentration (**b**) compared with the reference standard for each phantom and exposure setting. HU = Hounsfield unit, mAs = quality reference mAs, SFW = sand + flour + water

**Fig. 4:**
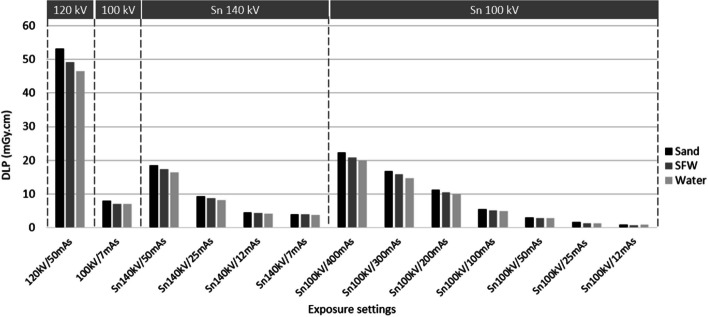
DLP associated with exposure settings for the three phantoms. DLP = dose length product, mAs = milliampere seconds (quality reference), SFW = sand, flour, water

Figure 3b shows a negligible effect on PET quantification (≤ 1.7%) for all three phantoms, with the quantification difference increasing with density. This provided a DLP of 7–8 mGy.cm for the 1 bed position PET-CT scans, at 85% lower dose than the 120 kV/50 mAs-ref reference (Fig. 4).

### Sn100 kV

**Fig. 5:**
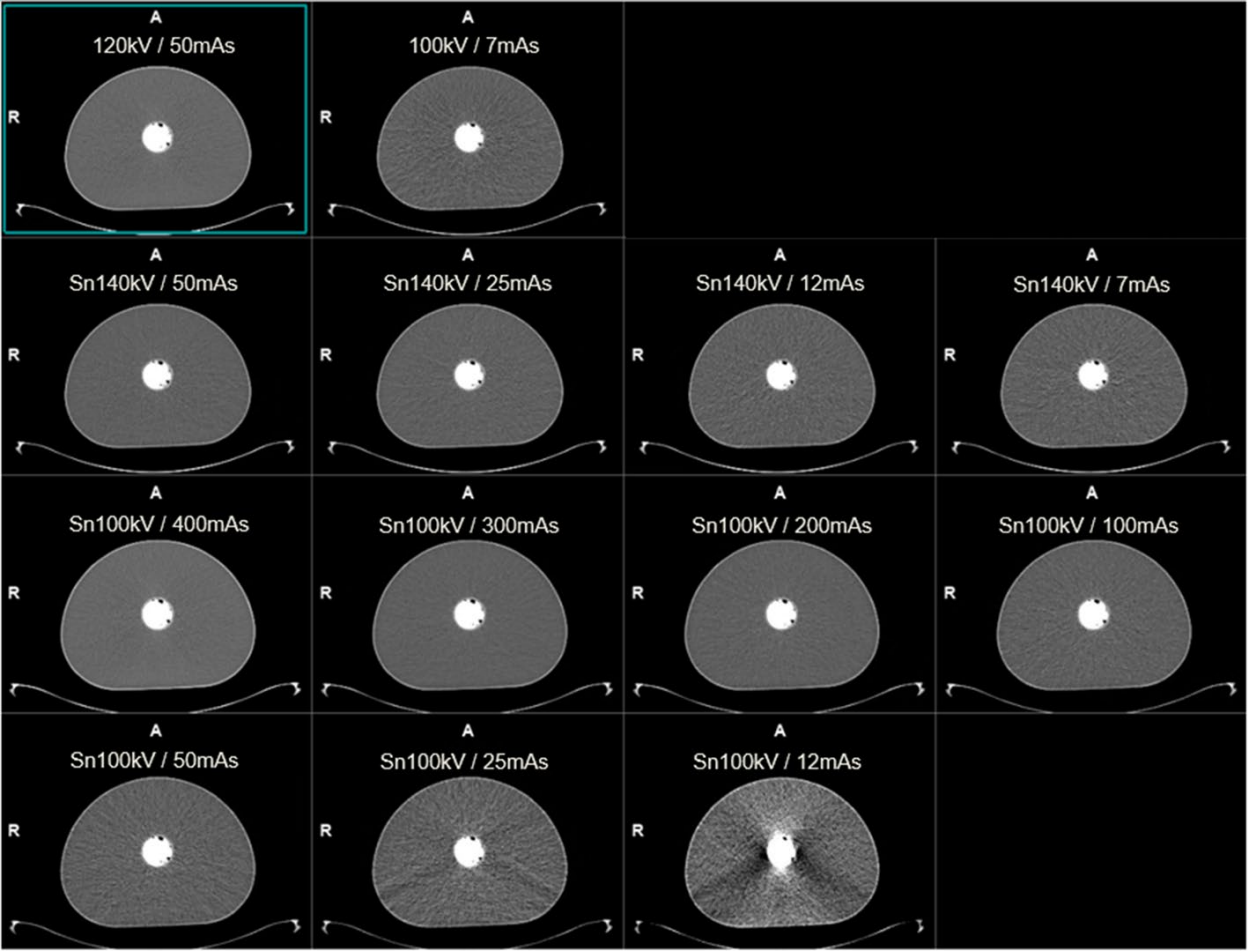
Transaxial slice of sand phantom at each exposure setting showing CT artefacts at lower photon fluence

All mAs settings down to 50 mAs-ref provided negligible effect on PET quantification in all three phantoms (≤ 1.2%) (Fig. 3b), and CT HUs were similar to those of the 120 kV reference (Fig. 3a). At this setting, the DLP of 2.8 mGy.cm in water is 60% lower than that provided by the standard ULDCT for AC protocol (Fig. 4). In water, PET quantification differences remained negligible (≤ 1.7%) down to the lowest investigated value of 12 mAs-ref (Fig. 3b), providing a DLP of 0.9 mGy.cm, 87% lower than the dose for standard ULDCT for AC (Fig. 4). However, in bone-equivalent densities, differences in PET quantification exceeded 2% at 25 mAs-ref and below (Fig. 3b), consistent with a marked reduction in CT HU (Fig. 3a). Figure 5 shows the presence or absence of artefacts exhibited in the sand phantom at the different exposure settings, showing marked artefacts at Sn100kV/12mAs.

### Sn140kV

In water, PET quantification differences were negligible (≤ 1.4%) down to 7 mAs-ref (Fig. 3b), where the DLP of 3.8 mGy.cm provides a dose reduction of 46% compared with the standard ULDCT protocol for AC (Fig. 4). However, at bone equivalent densities, differences in PET quantification exceeded 2% (Fig. 3b).

## Discussion

### Summary and interpretation of findings

This study firstly demonstrated that the standard ULDCT protocol for PET AC without tin filter maintains adequate PET quantification at both soft tissue and bone equivalent densities (Fig. 3b), for a DLP of 7–8 mGy.cm per 26.3 cm bed position, when scanning a phantom representing a standard adult abdomen (Fig. 4). Given that few facilities have been using such low CT doses for PET AC only protocols [3], it is hoped that the findings of this study will encourage implementation of well-optimised ULDCT protocols when performing CT only for PET AC. Whilst differences in PET quantification with CT tube voltage changes had been previously investigated in soft tissue [15, 16], this study is the first to investigate this in bone equivalent densities, and should assist in optimisation of CT protocols for PET-CT examinations in which bone is a tissue of interest.

Moreover, this is the first study to examine the effect and benefits of the tin filter in PET-CT, demonstrating that it can further reduce dose in ULDCT scans performed only for PET AC by at least 60% (Fig. 4), whilst maintaining adequate PET quantification (Fig. 3b). The minimum required exposure settings, and therefore the dose savings which can be made, depend on the tissue density (Figs. 3b, 4) and accepted changes in PET quantification. At soft tissue equivalent, accurate PET quantification is maintained down to a DLP of 0.9 mGy.cm (Sn100 kV/12 mAs-ref) for the single bed position phantom scan (Fig. 3b), providing 87% dose reduction compared with the standard ULDCT protocol (Fig. 4). However, at bone-equivalent densities, higher exposure settings than this were required to achieve negligible differences (≤ 2%) in PET quantification (Fig. 3b), due to a marked reduction in measured CT HUs (Fig. 3a). This is likely due to a combination of beam hardening artefact from the large thickness of high-density material in the cylindrical insert as shown in Fig. 5, and excessive noise providing a bias in CT data, from absent signal in parts of the sinogram [17, 18]. There is no consensus as to what differences in PET quantification are acceptable, and the lower dose option could be used if differences need not be negligible but clinically acceptable, at ≤ 5% for example.

Sn100 kV provided the best compromise between PET quantification accuracy and dose compared with Sn140 kV (Figs. 3b, 4), given that the beam energies with Sn100 kV provide CT HUs similar to 120 kV with standard filtration (Fig. 3a), and the large effect of increased CT tube voltage on dose. Interestingly, a negative difference in absolute HU corresponded to a positive difference in PET activity concentration for Sn140 kV but not for Sn100 kV. This phenomenon can be explained by the bilinear transformation curve used to derive LAC from CT HU [2]. In this bilinear transformation, at less than around 50 HU LAC is not dependent on tube voltage, but above this HU threshold, LAC is dependent on tube voltage, with a higher kVp giving a higher LAC. Thus, for a given difference in HU compared with the reference standard CT, a higher PET quantification value is seen for Sn140kV compared with Sn100kV, in the bone-equivalent density phantoms.

Whilst this study focused on investigation of ULDCT protocols, data were also gathered for the tin filter at comparable doses to standard localisation CT, with Sn100 kV/400 mAs giving comparable dose to 120 kV/22 mAs. Hence, this work has also validated that PET quantification with the tin filter is also acceptable at higher doses.

### Clinical implementation

PET AC is the primary clinical purpose of the CT scan in cardiac and brain PET-CT examinations [3, 19]; hence, the study findings can be used to enable optimisation of CT radiation dose in these widely performed examinations. In addition, ULDCT scans are also performed when a diagnostic CT scan has already been recently acquired for the patient, or when multiple CT scans are performed in a PET-CT protocol, such as in multiparametric PET [20]. Furthermore, the dose reduction in ULDCT protocols afforded by the tin filter would be particularly beneficial for imaging with long axial field-of-view (LAFOV) PET-CT systems, since the CT scan must always cover the entire PET FOV. Yet, sometimes just a single organ is of interest. Furthermore, owing to their high PET sensitivity, LAFOV systems are used to scan children [21] and pregnant patients [22] allowing PET dose reduction, whom would also benefit from accompanying ULDCT for PET AC. LAFOV systems also scan many research participants and scan patients at multiple timepoints [21], for which an additional effort should be made to keep radiation doses as low as reasonably achievable.

When implementing ULDCT protocols, it is important to use a fast rotation time and high pitch factor, to allow the system to provide a lower effective mAs when delivering the lowest available tube current [23], since the lowest possible effective mAs is determined by the product of the tube current and rotation time (mAs) divided by the pitch value. An additional benefit of tin filter imaging for ULDCT is that a higher reference mAs is needed compared with standard filtration. This means that when tube current modulation is applied, for small patients and low-density body regions, the mAs can go lower than the reference as required, whereas the standard ULDCT protocol is already set to deliver just 7mAs-ref, and the tube cannot use a much lower current.

### Study design and future work

A ± 2% difference in PET quantification compared with the reference standard was considered negligible by the authors in this study, although there are no guidelines to inform this decision. However, since it had been reported that variation in standardised uptake value (SUV) maximum, SUV_max_, can exceed 15–20% in clinical practice [24] and its value had thus been debated for many years [25, 26], this seemed like a reasonable compromise between allowing some additional error on the SUV measurement which is already subject to considerable inaccuracy, whilst not increasing the error so much that its clinical or research utility is brought further into question. However, whilst we considered a ± 2% difference in PET quantification to be negligible, this does not mean that larger differences in PET quantification would not be clinically acceptable. This topic should be discussed further in relation to the specific clinical or research circumstances under which the data are used, for example, the trade-off between absolute effective dose saving and quantification accuracy, different patient groups, and how the quantitative value will be used.

The CT acquisitions undertaken in this study used the spine organ characteristic, since that was in clinical use for bone PET-CT examinations and the phantoms with bone-equivalent densities in the cylindrical insert represented imaging of a spine. It should be borne in mind that use of other organ characteristics will deliver slightly different effective mAs for a given reference, hence to deliver the same dose and image quality, a slightly different quality reference mAs may be required.

The CT images were reconstructed with ADMIRE, which is the best available CT reconstruction on Siemens Healthineers PET-CT systems, providing superior artefact reduction to the other types. Other types of CT reconstruction may provide slightly different artefact severities and CT HUs, thereby yielding slightly different PET quantification results. However, Definition Edge CT systems with tin filter are usually equipped with ADMIRE.

This study evaluated ULDCT scans in a standard abdominal-sized phantom. CARE Dose 4D tube current modulation was utilised for this phantom investigation, since this is normally applied to patient scans in clinical practice, to ensure consistent image quality for all patient sizes, as well as in all slices across the z-axis despite differing organ densities. Henceforth, the findings in this study should also be applicable to obese patients. However, a greater number and severity of artefacts are expected in larger patients, especially in slices where highly attenuating structures are present, for example between the shoulders and hips, which are particularly susceptible to beam hardening artefacts. This could change CT HUs dramatically in some locations, potentially affecting PET quantification. An additional iterative beam hardening correction has recently been added to ADMIRE, which may yield slightly different results, although this has not been investigated in this study. On the other hand, the thickness of the high-density sand in the cylindrical insert might overestimate the extent of beam hardening in a patient. Thus, to help determine the most appropriate exposure settings for tin filter ULDCT for PET AC, future work should examine the extent and location of CT artefacts in standard and obese-sized patient phantoms at the different exposure settings, and also quantify the effect of CT HU differences on PET quantification on the Biograph Vision.

Since TOF performance influences how robust PET reconstructions are to data inconsistencies [27], it should be considered that these data were gathered on a Biograph Vision silicon photomultiplier (SiPM) PET system, with 249 ps timing resolution [28]. It could also be investigated as to how PET quantification is affected with the examined protocols on conventional photomultiplier PET systems such as the Biograph mCT Edge, with slower timing resolution at 540 ps [29], if equipped with the tin filter, or if the standard ULDCT protocol is used for AC only.

Whilst this study has focused on the tin filter in ULDCT for PET AC only, CT scans are also commonly carried out for lesion localisation and characterisation, requiring superior image quality and therefore increased dose. Future work should evaluate how much CT dose reduction is afforded with the tin filter for localisation/characterisation CT for the different exam types, and which exposure settings are required.

This study has only considered CT tin filtration on PET-CT systems, yet the tin filter has also been recently introduced to single-photon emission computed tomography (SPECT)-CT. Although we would still expect to see marked dose savings with the tin filter in SPECT-CT, the magnitude of dose saving may be different, since these systems use different tube voltages, and the effect of HU changes may affect SPECT quantification to a different extent. Hence, a similar study should also be conducted for SPECT-CT to enable optimisation of tin filter protocols on those systems.

## Conclusions

This study has demonstrated that adequate PET quantification is maintained at the full range of tissue densities when using the standard ULDCT protocol for PET AC only, without tin filter on the Biograph Vision with Definition Edge CT, for a DLP of 7–8 mGy.cm per bed position. Moreover, using the tin filter could provide a further 60–87% dose reduction, with the minimum required CT dose depending on the tissue of interest and the accepted level of change in PET quantification. PET quantification accuracy is maintained in soft tissue equivalent density at a lower dose than in bone equivalent density. For ULDCT for PET AC, Sn100 kV provides the best compromise between dose and PET quantification accuracy compared with Sn140 kV. The optimal mAs-ref setting for Sn100 kV in ULDCT for PET AC lies between 12 and 50, but future work is needed to examine the effect of ULDCT protocols on beam hardening artefacts at typically affected anatomical sites, and examine their effect on PET quantification, in order to establish the optimal setting.

## Data Availability

The datasets used and/or analysed during the current study are available from the corresponding author on reasonable request.

## Abbreviations

^18^F-FDG: Fluorine-18 labelled fluorodeoxyglucose
AC: Attenuation correction
ADMIRE: Advanced modeled iterative reconstruction
CT: Computed tomography
DLP: Dose length product
FOV: Field-of-view
HU: Hounsfield units
kV: Kilovolt
LAC: Linear attenuation coefficient
LAFOV: Long axial field-of-view
mAs: Milliampere seconds
mAs-ref: Image quality reference used in tube current modulation [(tube current*rotation time)/pitch]
MBq: Megabecquerel
NEMA IEC: National Electrical Manufacturers Association/International Electrical Commission
PET: Positron emission tomography
SFW: Sand + flour + water
SiPM: Silicon photomultiplier
Sn: Symbol for the chemical element tin
SPECT: Single-photon emission computed tomography
SUV: Standardised uptake value
SUV_max_: Standardised uptake value maximum
TOF: Time-of-flight
ULD: Ultra-low-dose
VOI: Volume of interest
